# Generative Adversarial Networks (GANs) in the Field of Head and Neck Surgery: Current Evidence and Prospects for the Future—A Systematic Review

**DOI:** 10.3390/jcm13123556

**Published:** 2024-06-18

**Authors:** Luca Michelutti, Alessandro Tel, Marco Zeppieri, Tamara Ius, Edoardo Agosti, Salvatore Sembronio, Massimo Robiony

**Affiliations:** 1 Clinic of Maxillofacial Surgery, Head-Neck and NeuroScience Department, University Hospital of Udine, p.le S. Maria della Misericordia 15, 33100 Udine, Italyalessandro.tel@icloud.com (A.T.);; 2 Department of Ophthalmology, University Hospital of Udine, Piazzale S. Maria della Misericordia 15, 33100 Udine, Italy; 3 Neurosurgery Unit, Head-Neck and NeuroScience Department, University Hospital of Udine, p.le S. Maria della Misericordia 15, 33100 Udine, Italy; 4 Division of Neurosurgery, Department of Medical and Surgical Specialties, Radiological Sciences and Public Health, University of Brescia, Piazza Spedali Civili 1, 25123 Brescia, Italy

**Keywords:** generative adversarial network (GAN), artificial generating content, maxillofacial surgery, otolaryngology surgery

## Abstract

**Background:** Generative Adversarial Networks (GANs) are a class of artificial neural networks capable of generating content such as images, text, and sound. For several years already, artificial intelligence algorithms have shown promise as tools in the medical field, particularly in oncology. Generative Adversarial Networks (GANs) represent a new frontier of innovation, as they are revolutionizing artificial content generation, opening opportunities in artificial intelligence and deep learning. **Purpose:** This systematic review aims to investigate what the stage of development of such technology is in the field of head and neck surgery, offering a general overview of the applications of such algorithms, how they work, and the potential limitations to be overcome in the future. **Methods:** The Preferred Reporting Items for Systematic Reviews and Meta-Analyses (PRISMA) guidelines were followed in conducting this study, and the PICOS framework was used to formulate the research question. The following databases were evaluated: MEDLINE, Embase, Cochrane Central Register of Controlled Trials (CENTRAL), Scopus, ClinicalTrials.gov, ScienceDirect, and CINAHL. **Results:** Out of 700 studies, only 9 were included. Eight applications of GANs in the head and neck region were summarized, including the classification of craniosynostosis, recognition of the presence of chronic sinusitis, diagnosis of radicular cysts in panoramic X-rays, segmentation of craniomaxillofacial bones, reconstruction of bone defects, removal of metal artifacts from CT scans, prediction of the postoperative face, and improvement of the resolution of panoramic X-rays. **Conclusions:** Generative Adversarial Networks may represent a new evolutionary step in the study of pathology, oncological and otherwise, making the approach to the disease much more precise and personalized.

## 1. Introduction

### 1.1. Background

For several years now, artificial intelligence (AI) algorithms have been a topic of strong interest. Scientific studies in the literature are showing how these algorithms are excellent and promising tools that can be applied in various fields, particularly in the medical field. Artificial intelligence models are gaining more and more importance especially in oncology. Indeed, through the analysis of histologic, radiomic, genomic, and clinical–epidemiological data, these algorithms are proving to be potential tools for the detection of precancerous lesions, for diagnosis, for staging, for predicting response to treatment, and for the prognostic evaluation of a tumor [1,2,3,4,5].

**Figure 1:**
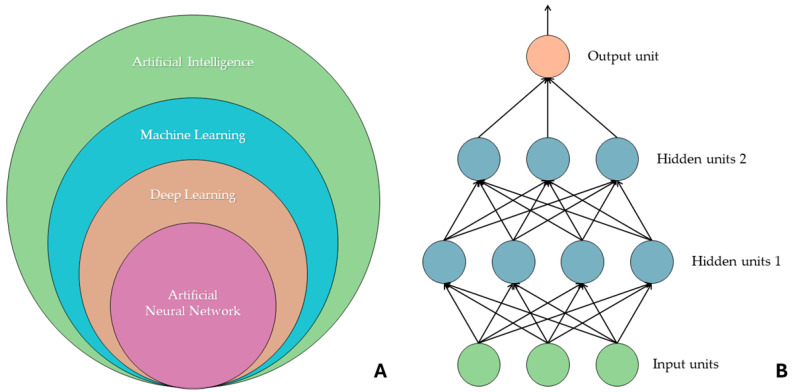
(**A**) Hierarchy between machine learning, deep learning, and artificial neural networks. (**B**) The composition of an artificial neural network consisting of an input layer (in green), two hidden layers (in blue), and an output layer (in orange) is shown. Each output within the ANN is used as input for the next layer. These artificial intelligence models, to perform the task for which they are trained, require a significant amount of data for the training set, particularly CNNs. In fact, there are studies in the literature that report the size of the data sample analyzed as a limitation. For example, the study conducted by Romeo et al. (2020) showed how the application of radiomic ML to primary tumor lesions has great potential in predicting the lymph node status of patients with oral cavity and oropharynx lesions, but they report how the small sample size is a problem. This is just one of many promising studies in the literature that report a similar impediment in evaluating the effectiveness and accuracy of such artificial intelligence models [10].

“Artificial intelligence”, “machine learning”, “deep learning”, and “artificial neural network” are often used as synonyms, but they refer to different concepts. The widest semantic area includes AI, which includes machine learning, and, on a further subdivision level, there are other models, including deep learning and artificial neural networks. ML is a field of artificial intelligence that focuses on learning through data input, and there are four kinds of learning methods: reinforcement learning and supervised, semi-supervised, and unsupervised learning. DL models are inspired by neural networks present in human beings. They are artificial neural networks that allow multilevel computational models to learn data representations with different and various levels of abstraction. In fact, there is an analogy with the cell bodies of a neuron, i.e., the nodes of the neural network, and axons, i.e., the connections that are generated between different nodes. An ANN, called a feedforward neural network, consists of an input layer and an output layer, and in between these are several hidden layers (which, in deep ANNs, can be dozens or hundreds in number). Information passes from each node in one layer to the next, and during this step the information is processed and transformed. As the input proceeds through an ANN, it is transformed so that when it reaches the final layer, it is no longer the same as its initial state. A particular type of ANN is the convolutional neural network (CNN), which sees wide application in image processing [6,7,8,9]. Figure 1 graphically describes the hierarchical organization of artificial intelligence networks.

To overcome the problem related to limited sample size, especially when studying rare diseases for which clinical data are complex, researchers are investigating the capabilities of Generative Adversarial Networks (GANs) as a tool to generate synthetic data, i.e., created by the AI model, in order to use them to train deep learning algorithms [11].

### 1.2. Generative Adversarial Network (GAN)

Although GANs have become the subject of strong interest only in recent years, these models were first introduced in 2014 by Ian J. Goodfellow et al. [12] during their doctoral work at the University of Montréal.

**Figure 2:**
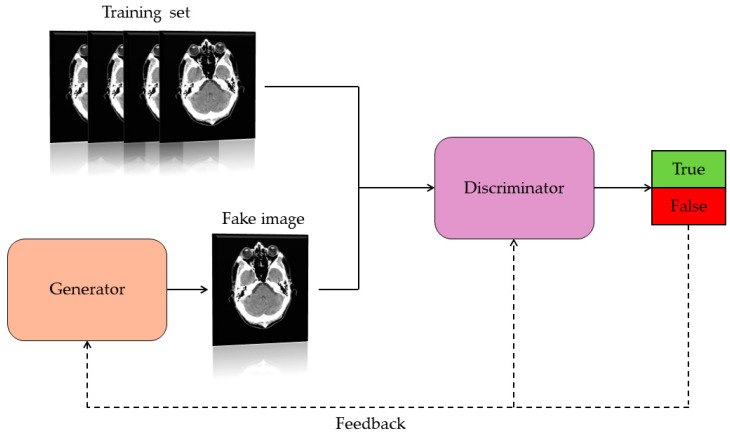
Basic architecture operation of a GAN.

Generative Adversarial Networks are a class of artificial neural network capable of creating artificial content such as images. Precisely, they consist of two networks: the generator and the discriminator (Figure 2). These have the task of competing with each other. Specifically, the generator must learn the data distribution and generate artificial data by receiving a random vector as input. The discriminator is trained to discriminate the difference between true data and synthetic data. Specifically, the discriminator receives synthetic and real data and calculates the probability that the generated data is real or false. The value of this probability represents a feedback signal that is sent to the discriminator itself and to the generator. This interactive process between the two networks (generator and discriminator) results in continuous mutual improvement, resulting in images similar to the real ones. The goal of a GAN is to generate synthetic images that are indistinguishable from real ones [11,13].

From 2014, when GANs were born, to the present, these artificial neural networks have undergone continuous evolutions that have given birth to new and numerous architectures, some specific to certain functions (Table 1) [13,14].

### 1.3. Objective of the Study

Already, several studies have shown how GANs are promising tools, especially to overcome the problem of the scarcity of clinical data for given diseases. For example, the study conducted by Loey et al. (2020) presented a GAN capable of generating synthetic data for coronavirus detection in chest X-rays [15], or the study conducted by Fujioka et al. (2020) reported the use of a GAN to create a computational model capable of detecting abnormal lesions in breast ultrasound images [16].

These models can represent a useful tool for the work of doctors and healthcare professionals. For example, the study conducted by Maniaci et al. (2024) [17] reported how the use of ChatGPT can be a tool capable of providing support in the diagnostic process in imaging for pathologies of the head and neck region. Furthermore, ChatGPT is proving to be a potential means to strengthen the relationship between doctor and patient, helping the latter to better understand their health status.

Considering that GANs have already been applied in different areas of medicine and in different pathologies, oncological and otherwise, the aim of this study is to propose an overview of what the applications of generative adversarial networks in pathologies affecting the head and neck region are, to report the reasons why researchers have been studying these particular artificial neural networks, to describe the types of GAN architectures used, and to analyze the results obtained from the studies included in this paper. Given that this is a technology that is gaining ground recently and considering that it is demonstrating promising applications in the management and study of many pathologies, we believe that it is interesting to investigate what the current applications and the possible prospects of such artificial intelligence models are.

## 2. Materials and Methods

This study was performed according to the Preferred Reporting Items for Systematic Review and Meta-Analyses (PRISMA) statement [18]. The PRISMA checklist is inserted in the Supplementary Materials. This review was recorded in the PROSPERO database (International Prospective Register of Systematic Review), and the ID number is 523954. The research question of this systematic review was constructed according to the PICOS framework (Table 2) and can be explained as follows: “Which are the applications of Generative Adversarial Networks for pathologies affecting the head and neck region?”.

### 2.1. Literature Search

The query used is: ((GAN) OR (generative adversarial network)) AND ((maxillofacial surgery) OR (otolaryngology surgery) OR (head neck surgery)). The MeSH query used to improve and extend the search is the following: ((“Artificial Intelligence” [Mesh]) OR “Neural Networks, Computer” [Mesh]) AND “Orthognathic Surgical Procedures” [Mesh]. Using keyword combinations, the literature search was conducted until 13 March 2024, searching for studies included in the following databases: MEDLINE, Cochrane Central Register of Controlled Trials (CENTRAL), ClinicalTrials.gov (accessed on 29 April 2024), ScienceDirect, Embase, Scopus, CINAHL. The types of included articles are clinical trials, randomized clinical trials, cohort studies, original articles, research articles, and also reviews, systematic reviews, and meta-analysis since there are few papers in the literature concerning this topic.

**Figure 3:**
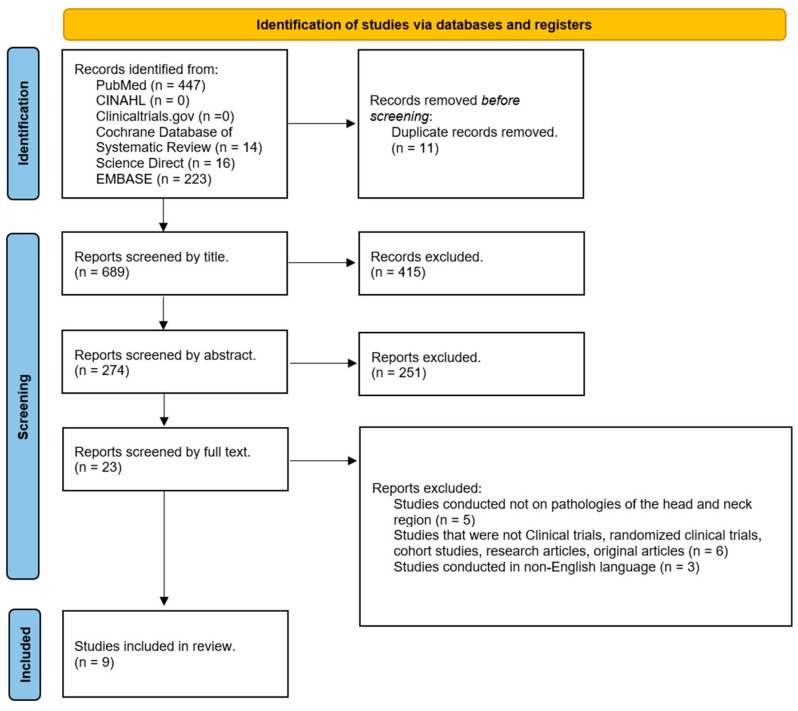
PRISMA flowchart of the systematic review process.

The articles found in the databases consulted were imported into EndNote21 (Clarivate, Analytics, Philadelphia, PA, USA). All articles exported to EndNote21 were screened by two separate investigators (L.M. and A.T.). The primary screening consists of evaluating first the titles and then the abstracts of the included studies, as reported in the PRISMA flowchart (Figure 3), and in case of doubt, a third investigator (M.R.) was included in the evaluation.

### 2.2. Data Collection

The data extracted from the studies included in this systematic review are study topic, reasons that motivated the researchers to study the application of GANs, architecture of GAN used, type of data used, parameters adopted to measure the functioning of the GANs, and the results obtained from the individual studies included. The data were manually exported by the two independent researchers (L.M and A.T.) and collected in a Microsoft Excel 2019 spreadsheet. These data are displayed in Table 3 of Section 3.

### 2.3. Inclusion and Exclusion Criteria Applied in the Collected Studies

The two researchers independently (L.M. and A.T.) applied the inclusion and exclusion criteria below on the collected articles through an initial evaluation on the title and abstract and then through a thorough analysis of the full text of the articles.

Articles analyzing the application of GANs in any pathology, oncologic or otherwise, of the head–neck region were considered. Articles dealing with GANs applied in pathologies not of the head–neck region, articles without the abstract, and systematic reviews and meta-analyses were excluded. Only studies conducted on the human species and published in English were included. The articles considered in this study are all papers that have a publication date between 2014 and 2024, and the types of studies included are clinical trials, original articles, cohort studies, and research articles.

### 2.4. Bias Assessment

**Figure 4:**
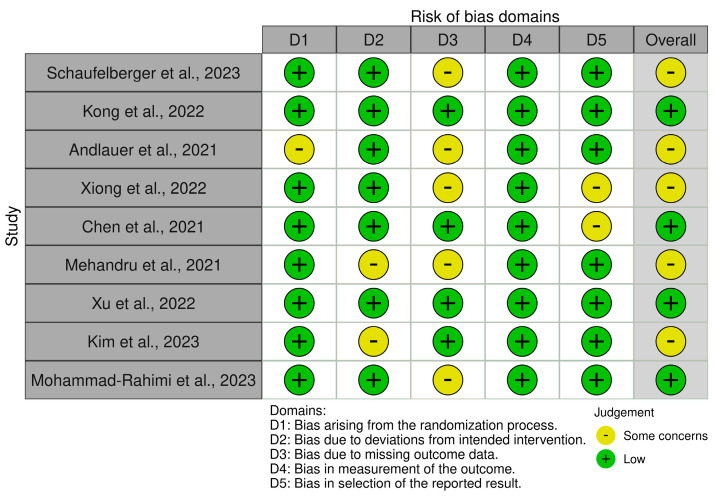
Robvis tool for assessing the risk of bias.

Five types of bias were assessed: bias arising from the randomization process, bias due to deviations from the intended interventions, bias due to missing outcome data, bias in outcome measurement, and bias in reported outcome selection (Figure 4). The Robvis tool [19] was used to analyze the presence of these biases, and two investigators (L.M. and A.T.) were involved, who worked independently.

## 3. Results

PRISMA guidelines were followed in the search for scientific evidence to be included in this study. Figure 3 shows the PRISMA flowchart that illustrates the entire process of article selection. The total number of articles collected from the databases consulted and exported to EndNote is 700. Of these, 11 were removed because they were dubbed. Of the 689 remaining, 415 were excluded by analyzing the titles of the studies (although some collected studies did not have the word “GAN” in the title, the abstracts were carefully analyzed before deciding whether or not to exclude these studies). Screening based on abstract analysis resulted in the elimination of 251 reports. Of the remaining 23 studies, after careful and attentive analysis of the complete texts, 9 studies remained.

Through the use of the Robvis tool [19], the biases of the nine included studies were analyzed. Figure 4 illustrates the results obtained from this assessment.

### 3.1. Topics

**Figure 5:**
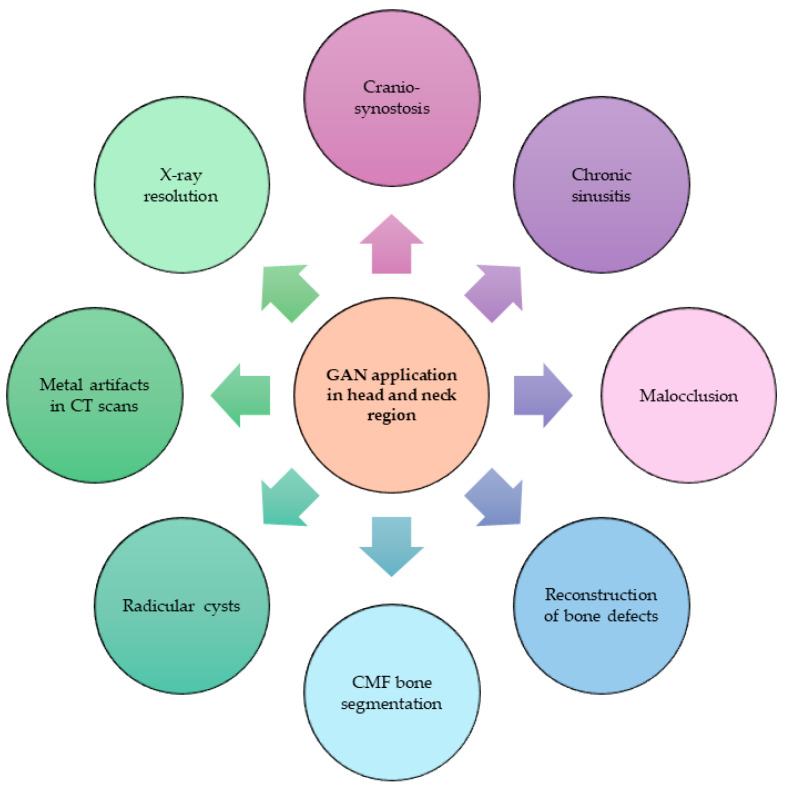
Different applications of GANs for the head and neck region.

Eight different types of topics were covered in the studies included in this paper (Figure 5): classification of craniosynostosis, chronic sinusitis, temporomandibular disorders and malocclusion, reconstruction of bone defects, craniomaxillofacial (CMF) bone segmentation, radicular cysts, correction of metal artifacts in CT scans, and improvement of panoramic X-ray resolution.

### 3.2. Reasons Why GANs Have Been Applied

One of the most common reasons for researchers to study and apply GANs is the scarcity of clinical data. As repeated many times, artificial intelligence algorithms, especially artificial neural networks, need a large amount of data to be trained in order to perform the task for which they were created. The difficulty in finding clinical data is attributed to multiple factors that make obtaining such data complex: the presence of a rare disease for which little information and data are available; the need for experienced radiologists; the use of scans and images with specific characteristics in terms of format and source; and privacy issues.

In addition to obviating the problem of clinical data retrieval by generating synthetic data identical to real data, GANs have also been applied for other reasons, including the ability to overcome the limitations of current methods of reconstructing midfacial bone defects, translation of a CT scan into an MRI image, removal of metal artifacts, and improvement of the resolution of panoramic X-rays by exploiting the denoising function.

### 3.3. GAN Architectures Used

In the included studies, we have reported several types of GAN architectures, including cDC-WGAN-GP (Conditional Deep Convolutional Wasserstein GAN with Gradient Penalty), AC-GAN (Auxiliary Classifier GAN), Cycle-GAN, DDA-GAN (Diverse Data Augmentation Generative Adversarial Network), CD-GAN (Cyclic Discriminative GAN), MAR-GAN (metal artifact reduction GAN), Pix2pix-GAN, and SR-GAN (Super-Resolution GAN). Their functioning is briefly illustrated in Table 3.

**Table 3:** Functioning of the GAN architectures of the included studies.

cDC-WGAN-GP	It is a model that combines the Wasserstein GAN (WGAN) and the Gradient Penalty (GP). The former can produce better quality samples, the latter introduces a penalty on gradients preventing them from vanishing or exploding further enhancing stability. In addition, being a conditional model, it can generate specific images, resulting in an advanced model for synthetic image generation.
AC-GAN [20]	It is an extension of conditional GAN (cGAN), which in turn is an extension of the GAN architecture. The cGAN can predict the class label of an image received as input. The AC-GAN has a discriminator that predicts the class label of an image.
Cycle-GAN [21]	It is an image–image translation model without the need to have paired examples. By image–image translation, we mean the creation of a new artificial version of an image with specific modifications.
DDA-GAN [22]	It is a model that can segment bone structures and exploit synthetic data generated from an annotated domain to improve the quality of segmentation of images from an unannotated domain.
CD-GAN [23]	It is a model for image-to-image transformation, transforming an image from one domain to another. It is based on a Cycle-GAN architecture, but unlike the latter, it evaluates the quality of synthetic images by additional cyclic discriminators, making them more realistic.
MAR-GAN	GAN model capable of removing metal artifacts present in CT scans.
Pix2pix-GAN [24]	It is a model of cGAN (conditional generative adversarial network) used for image-to-image translation. It features a generator that is based on the U-Net architecture and a discriminator represented by a PatchGAN classifier.
SR-GAN [25]	It is a model used for super-resolution imaging. It has the function of generating high-definition images from low-resolution images.

### 3.4. Type of Data Analyzed

The data that were used were exclusively imaging data. In terms of percentages, in 44.4% of the included studies panoramic X-rays were used, 55.5% used CT scans, and 11.1% used MRI images.

Some studies are not limited in using only one type of imaging data. The study conducted by Kong et al., 2022 [26] exploits both CT and paranasal RX scans, the study conducted by Andlauer et al., 2021 [27] crosses both 2D images and postoperative 3D simulated images obtained from processing CT scans, and the study conducted by Chen et al. [28] analyzes both CT and MRI scans.

### 3.5. Parameters Adopted to Evaluate GANs

In the included studies, the parameters used to evaluate the results obtained are F1 score, AUC (area under the curve), sensibility, specificity, PSNR (peak signal-to-noise ratio), Euclidean distance, DSC (coefficient of symmetry), average symmetric superficial distance, SSIM (structural similarity index measurements), MSE (mean square error), PPV (positive predictive value), NPV (negative predictive value), and MOS (mean for expert opinion score).

### 3.6. Results Obtained for the Single Studies Included

In the study conducted by Schaufelberger et al., 2023 [29] a CNN (convolutional neural network) was trained to classify the different types of craniosynostosis using only synthetic data generated by different generative models, including GANs. The CNN was able to grade four different types of deformity.

The study conducted by Kong et al., 2022 [26] also demonstrated how GANs are an effective source of data that can be exploited to train artificial intelligence models to perform the job for which they were created. Through a particular type of GAN, synthetic data were generated to train a deep learning model created to diagnose the presence of sinus pathology by studying panoramic RX images and CT scans. It was shown that the deep learning model had better diagnostic performance when trained with original data and synthetic data generated GAN data.

The study conducted by Mehandru et al., 2021 [30] also demonstrated how GANs can generate useful data to train a CNN to recognize the presence of root cysts by studying panoramic X-rays. Analyzing the ROC curve, the CNN trained without synthetic images had lower accuracy than the CNN trained with synthetic images created by GANs (89.3% vs. 95.1%, respectively). The study conducted by Andlauer et al., 2022 [27] used a Cycle-GAN to predict the postoperative face of a patient with class II and III malocclusion to undergo bimaxillary surgery. Using 2D images and a 3D simulation of the surgery, the GAN was able to predict the outcome of the surgery. The Cycle-GAN was shown to predict realistic chin and nose changes on selected examples.

The study conducted by Xiong et al., 2022 [31] used a GAN to reconstruct midfacial bone defects. On the real and normal CT scans, spherical, cuboid, and semi-cylindrical artificial defects were manually inserted in five structural regions to simulate the bone defects. To train GANs, scans with corresponding artificially created defects were important. By analyzing the cosine similarity, this was about 0.97 in the reconstruction of artificially created defects and 0.96 in the reconstruction of unilateral clinical defects.

GANs also show promise for segmentation of craniomaxillofacial bones, as shown in the study conducted by Chen et al., 2021. [28] The GAN architecture used in the study is the DDA-GAN, which was compared to other segmentation tools, including PnP-AdaNet, SIFA, and SynSeg-Net. The DDA-GAN outperformed SynSeg-Net by 2.68 in terms of Dice symmetry coefficient (DCS) and 0.13 in terms of average surface symmetric distance (ASSD).

The study conducted by Xu et al., 2022 [32] applied a particular type of GAN, a MAR-GAN, to eliminate metal artifacts present in CT scans. The MAR-GAN performed better, demonstrating excellent abilities in restoring the original structures near metals and removing metal artifacts.

The two included studies that applied GANs to improve the resolution of panoramic X-rays, Kim et al., 2023 and Mohammad-Rahimi et al., 2023 [33,34] showed promising performance. The first study used a Pix2Pix-GAN model, demonstrating good performance on images with blurring in the anterior tooth region, while the second study used an SR-GAN model, demonstrating in terms of MOS (mean opinion score) significant improvements in resolution compared to the other tools used and compared.

Table 4 provides a comprehensive overview of papers selected for this systematic review.

## 4. Discussion

As announced earlier in the introduction, this systematic review aims to present what the applications of GANs (Generative Adversarial Networks) in pathologies are, oncological and otherwise, affecting the head and neck region. GANs are proving to be very promising tools in the field of medicine and with multiple functions. One of the most important functions is the generation of synthetic images. In fact, the creation of artificial data is solving a problem that many studies have raised, namely the scarcity of clinical data to be used to train deep learning models developed to perform the different functions for which they were created. As we have also seen in this paper, to train a deep learning model requires a significant amount of clinical data, and often this is not always possible, for various reasons, including the need for experienced radiologists, issues related to patient privacy, and use of data with particular properties that are often not always the same, especially in multicenter studies that analyze data from multiple different centers using different modalities to obtain CT or MRI scans.

In this paper, we have reported eight applications of GANs for pathologies affecting the head and neck region: classification of craniosynostosis, recognition of the presence of chronic sinusitis, diagnosis of radicular cysts in panoramic X-rays, segmentation of craniomaxillofacial bones, reconstruction of bone defects, removal of metal artifacts from CT scans, prediction of the postoperative face, and improvement of the resolution of panoramic X-rays. In these areas, GANs have proven essential for their ability to generate large amounts of synthetic data and more. In fact, depending on the type of architecture, GANs can acquire multiple functions, including denoising, image translation, reconstruction, segmentation, and classification.

Unfortunately, we did not find any studies dealing with GANs applied in the field of head and neck cancers, although there are several papers in the literature analyzing the application of GANs for functions that are certainly useful in oncology, such as gene expression analysis, segmentation, tumor detection, and diagnosis. There are several examples of how GANs can be implemented in the study of cancer pathology. A recent study by Waters et al., 2024 [11] investigated the use of GANs for augmented gene expression analysis and demonstrated how this technology can reliably discover gene expression in a limited number of samples, proving to be extremely useful, especially for rare diseases for which little clinical data are available. Also, in the field of oncology, the study conducted by Park et al., 2021 [35] applied a GAN to generate synthetic images in order to observe morphological changes in glioblastomas to improve the diagnostic performance of this pathology.

Since there are already several studies applying such technology in the oncological setting, we invite future research to investigate what the potential of GANs might be in head and neck cancer, particularly in oral cancer. Artificial intelligence applied to head-neck cancer pathology has been a subject of strong interest for several years already. It has been successful in demonstrating how machine learning and deep learning models are promising tools in various areas of oncology, including diagnosis, segmentation, staging, and even prognostic evaluation of cancer, as also shown by the systematic review conducted by Michelutti et al., 2023 [36] and the study by Chinnery et al., 2021 [37].

Although GANs are proving to be innovative tools, some limitations may exist, as the study conducted by Chen et al., 2021 [38] points out. Some of the problems concern mode collapse, i.e., when the generator is no longer capable of producing a large amount of artificial data; nonconvergence, i.e., when the generator produces more and more realistic data and the discriminator is no longer able to follow this evolution as the latter’s feedback becomes increasingly meaningless; diminished gradient, which is when the generator cannot improve its performance as fast as the discriminator; overfitting, which is when the amount of data is very limited; and imperfection, which is when no evaluation function can mimic human judgment.

These tools are showing great promise, but one must also consider that these tools, particularly ChatGPT, need to be evaluated and it is necessary to understand whether the performance is reliable. In this regard, the study conducted by Lechien et al. (2024) [39] applied an instrument, the Artificial Intelligence Performance Instrument (AIPI), to evaluate the performance of ChatGPT in the management of ENT patients. Although it has demonstrated good performance, which has also been validated by ENT surgeons, the authors highlight the need for further future studies to investigate the usefulness of such an instrument in medicine and surgery.

While we are aware that GANs applied in the medical field are an innovative and recently popularized topic, we must reiterate the absence of articles related to the study of this technology applied to head and neck oncology. We understand the reason why there are few reports on this subject in the literature, and precisely because the study of these artificial intelligence models is recent, we urge future research to conduct multicenter studies and protocols to standardize the application of GANs. We urge the study of such technology applied in the context of head and neck cancers. As mentioned above, many studies applying deep learning models in radiomics unfortunately have small patient samples and insufficiently large data sets. This technology could solve these limitations encountered. It is essential to investigate the impact that GANs can have in radiomics and in the study of CT and MRI scans of head and neck cancers so that we can have a useful tool in the study of oncological pathology through artificial intelligence algorithms. We believe that GANs can represent an important evolutionary step in the study of cancer, succeeding in overcoming the obstacles presented by deep learning models and making the transition to increasingly cutting-edge precision medicine faster and faster.

## 5. Conclusions

Generative Adversarial Networks (GANs) are artificial neural networks capable of performing many functions, particularly the generation of synthetic data. These artificial data are proving to be useful and essential for training deep learning algorithms, especially when dealing with a rare disease for which little clinical data are available. With regard to the application of GANs in the head and neck district, we found several areas where they are proving to be useful and promising tools, including classification of craniosynostosis, recognition of the presence of chronic sinusitis, diagnosis of radicular cysts in panoramic X-rays, segmentation of craniomaxillofacial bones, reconstruction of bone defects, removal of metal artifacts from CT scans, prediction of the postoperative face, and improvement of the resolution of panoramic X-rays. Unfortunately, no articles were found dealing with GAN applied in the specific study of head and neck cancer, although we believe that future research will be able to fill this hole. In conclusion, we believe that Generative Adversarial Networks may represent a new evolutionary step in the study of pathology, oncological and otherwise, making the approach to the disease much more precise and personalized.

## Figures and Tables

**Table 1:** GAN functions found in the literature.

Classification	Model that can correctly classify input data by only emitting labels. In this model, the discriminator is a classification network.
Segmentation	It is used to classify voxels in order to identify objects using two GANs: the first produces synthetic scans, while the second has a segmentation network (using synthetic and real data) and a discriminator.
Reconstruction	GANs trained to reconstruct incomplete objects through a process with the purpose of acquiring the form and appearance of real objects.
Denoising	GANs trained to rebuild the true appearance of the object by removal of noise, artifacts, and causal data.
Image translation	GAN capable of converting an input image into another artificial version of that image, for example, transforming a CT scan into an MRI image.
General activities	GAN capable of generating synthetic data without applying it in a specific activity.

**Table 2:** PICOS framework.

Participant	Patients with diseases, oncological and otherwise, of the head-neck region
Interventions	Evaluation of the effects of the application of GANs
Comparators	N/A
Outcomes	AUC, sensibility, specificity
Study design	Research articles, original articles, cohort studies, clinical trials, randomized clinical trials

**Table 4:** Data extracted by the included studies.

	Topics	Reason for Applying GANs	Architecture GAN Used	Data Used	Method	Results
Schaufelberger et al., 2023 [29]	Craniosynostosis classification	Generation of synthetic data to train a CNN to classify craniosynostosis, given the paucity of clinical data	cDC-WGAN-GP	75% of the clinical data were used to train GANs to generate synthetic data on which CNN was trained, while the remaining 25% was used for the test set for CNN.	A CNN was trained with purely synthetic data to classify craniosynostosis. The synthetic data were generated from three different generative models: GAN, SSM, and PCA.	Classification of craniosynostosis with a synthetic data set has been shown to have similar performance to that of a classifier trained on clinical data.
Kong et al., 2022 [26]	Imaging data generation for paranasal pathology (chronic sinusitis)	Synthetic data generation to improve the diagnostic performance of a DL model given the presence of possible problems in procuring clinical data, including: poor availability, need for experienced radiologists, and privacy issues	AC-GAN	Patients diagnosed with chronic sinusitis and undergoing X-ray examination were included. Included only patients who underwent both RX and CT with time distance of less than 14 days. The 389 paranasal RXs of the internal data set (212 images with sinusitis and 177 normal) were divided: 80% training set, 10% validation set, 20% test set).	All conventional radiographs were labeled as “sinusitis” or “normal” by experienced rhinologists by studying CT scans. One DL model, ChexNet, was trained in several ways, among them only with the original training data and with data obtained through GANs.	Of the various models analyzed in this study, the GBC + OMGDA model performed better in terms of AUC, accuracy, sensitivity, specificity, F1 score, PPV, and NPV. It was shown that the DL model had better diagnostic performance when trained with original data and synthetic data generated GAN data.
Andlauer et al., 2021 [27]	Postoperative prediction for temporomandibular disorders and malocclusion	Predicting the postoperative face using preoperative 2D images and 3D simulation of postoperative soft tissue, without the need for difficult-to-acquire preoperative 3D images, for patients to undergo bimaxillary surgery for class II and class III malocclusion	Cycle-GAN	Used preoperative 2D images and a postoperative 3D simulation using preoperative facial CT images of four patients with malocclusion	CT scans were used to simulate bimaxillary surgery, and bone and soft tissue segmentations were applied on being. These 3D simulations, along with preoperative 2D images, were used to train the GAN.	Cycle-GAN was shown to predict realistic chin and nose changes on selected examples. The accuracy of the predictions was evaluated by the Euclidean distance of the facial landmarks. Unfortunately, some prediction errors were found concerning the nose region, while there appeared to be no errors for the chin region. According to the authors, this is probably due to the need for more detailed plots for the study of the nose that change based on different head poses. Nevertheless, it seems to be a promising tool for predicting postoperative outcome.
Xiong et al., 2022 [31]	Reconstruction of midfacial bone defects	Training a GAN to reconstruct midfacial defects. There are reconstruction methods such as mirror technology, but it cannot be used for I midspan and bilateral defects. In addition, training the DL model requires a large amount of data, which is not always achievable with clinical data alone.	GAN	CT scans with different defects: median and unilateral were used in this study	For GAN training, spherical, cuboid and half-cylindrical artificial defects were manually inserted on the real and normal CT scans in 5 structural regions to simulate bone defects. To evaluate the performance of GAN, cosine similarity (an indicator to assess the similarity of two objects) was calculated. The reconstructed images were compared with the real ones, and the obtained effect of the rebuilt area was evaluated by surgeons.	To evaluate the performance of the GAN used, cosine similarity was assessed. The study obtained a result of 0.97 in terms of reconstruction of artificial defects and 0.96 in terms of reconstruction of unilateral defects.
Chen et al., 2021 [28]	Craniomaxillofacial bone segmentation	Synthetic data generation to train DL model to segment craniomaxillofacial CT scan. The paucity of available clinical data is a problem for having reliable segmentation models. In addition, we want to investigate whether from CT scans can be used to train segmentation models for MRI.	DDA-GAN	Use 50 CT scans as a training set and 50 MRI scans.	MRI-CT data were used to train the GAN to perform segmentations, and MRI scans were used for validation. The performance of the GAN was evaluated using DSC and ASSD, compared with different image segmentation methods, including PnP-AdaNet, SIFA, and SynSeg-Net.	The GAN analyzed by the study (DDA-GAN), outperforms SynSeg-Net, a Cycle-GAN used to train segmentation models. The DDA-GAN was shown to be superior because it avoids geometric distortions and improves segmentation performance.
Mehandru et al. 2021 [30]	Pathology detection in panoramic RX (root cysts)	Synthetic data generation to train a CNN to recognize root cysts in panoramic X-rays	CD-GAN	34 panoramic RXs of root cysts and 34 normal ones were divided into training and test sets (75% and 25% respectively). In addition to these, synthetic images generated by GAN were added.	Two CNNs were compared for the purpose of recognizing root cysts from panoramic X-rays: the first uses non-GAN-generated data, while the second also takes advantage of synthetic images. Both CNNs were then evaluated in terms of AUC.	The proposed model has demonstrated better performance in terms of area under the curve (AUC), sensitivity, and specificity. Comparing the receiver operating characteristic (ROC) curves, the performance of the convolutional neural network (CNN) trained with synthetic images was superior to that of the CNN trained with non-artificial images (95.1% vs. 89.3%).
Xu et al., 2022 [32]	Reduction of metal artifacts in oral maxillofacial CT	Build a GAN-based model to reduce artifacts and improve image quality	MAR-GAN	For this study, CT scans were used on which metal artifacts were artificially simulated.	The parameters used to evaluate the performance of the studied GAN are RMSE and SSIM.	The used GAN in this study has high performance, demonstrainting its ability to effictibely reduce the metallic artifacts that were artificially inserted.
Kim et al., 2023 [33]	Image resolution refinement of panoramic RX	Create a GAN model that can improve image quality	Pix2Pix-GAN	In this study, panoramic X-rays were used. From these, images with poor quality were obtained to test the Pix2Pix-GAN’s ability to improve image quality.	The quality of the generated images was evaluated by radiologist experienced in maxillofacial pathology.	The proposed model performed very well on images with blur in the anterior tooth region, while it was less effective in improving image quality with blur and noise.
Mohammad-Rahimi et al., 2023 [34]	Image resolution refinement of panoramic RX	Compare different DL-based super resolution (SR) models to improve the resolution of panoramic RXs	SR-GAN	888 Dental panoramic X-rays	Five super-resolution models were compared, including SRCNN, SRGAN, U-Net, Swinlr, and LTE. The results were compared with each other by conventional bicubic interpolation. The parameters of MSE, PNSR, SSIM, and MOS were evaluated.	The GAN included in this study (SR-GAN) has demonstrated significant performance in improving the resolution of panoramic X-rays in termins of mean opinion score (MOS).

Legends: AC-GAN—auxiliary classifier; ASSD—average symmetric superficial distance; AUC—area under the curve; CD-GAN—Cyclic Discriminative GAN; cDC-WGAN-GP—Wasserstein GAN and the Gradient Penalty; CNN—convolutional neural network; CT—computed tomography; DDA-GAN—Diverse Data Augmentation Generative Adversarial Network); DSC—Dice’s coefficient of symmetry; DL—deep learning; GAN—Generative Adversarial Network; LTE—local texture estimator; MAR-GAN—metal artifact reduction GAN; ML—machine learning; MOS—mean opinion score; MRI: magnetic resonance imaging; MSE: mean square error; NPV—negative predictive value; PCA—principal component analysis; PPV—positive predictive value; PSNR—peak signal-to-noise ratio; ROC—receiver operating characteristic; RMSE—root mean square error; RX—X-rays; SR—super-resolution; SRCNN—SR convolutional neural network; SSIM—structural similarity index measure; SSM—Statistical Shape Model; Swinlr—Swin for image restoration.

## Data Availability

Data are available in a publicly accessible repository.

## Supplementary Materials

The following supporting information can be downloaded at: https://www.mdpi.com/article/10.3390/jcm13123556/s1, PRISMA 2020 Checklist.
